# Gasdermin D Is a Novel Prognostic Biomarker and Relates to TMZ Response in Glioblastoma

**DOI:** 10.3390/cancers13225620

**Published:** 2021-11-10

**Authors:** Junhui Liu, Lun Gao, Xiaonan Zhu, Rongxin Geng, Xiang Tao, Haitao Xu, Zhibiao Chen

**Affiliations:** 1 Department of Neurosurgery, Renmin Hospital of Wuhan University, Wuhan 430060, China; doctorhsdqe@whu.edu.cn (J.L.); lungao@whu.edu.cn (L.G.); zhuxiaonan@gmail.com (X.Z.); rickygeng@whu.edu.cn (R.G.); lovecc@foxmail.com (X.T.); xuhaitaorenmin@163.com (H.X.); 2 Central Laboratory, Renmin Hospital of Wuhan University, Wuhan 430060, China

**Keywords:** gasdermin family, pyroptosis, glioma, prognosis, immune, temozolomide

## Abstract

**Simple Summary:**

GSMD family were crucial regulators of pyroptosis. We used WB, IHC and bioinformatics to explore expression and potential role of GSDMs family in the progression of GBM. We found that only GSDMD expression was upregulated in glioma compared with nontumor brain tissues both in the public datasets and in-house cohort. High GSDMD expression was significantly associated with WHO grade IV, IDH 1/2 wild-type, mesenchymal subtypes and shorter overall survival.Moreover, GSDMD expression increased with after treating with TMZ in a time-dependent manner. We conculded that GSDMD was a novel prognostic biomarker, as well as TMZ-treatment response marker in glioma.

**Abstract:**

The gasdermin (GSDM) family act as executioners during pyroptosis. However, its expression and biological role in glioma remain to be determined. This study carried out gene expression from six public datasets. Westerns blots and immunohistochemistry (IHC) staining were employed to examine GSDM expression in glioma in an in-house cohort. Kaplan–Meier and Cox regression analyses were performed to evaluate the prognostic role of GSDMs in glioma. Association between gene expression and immune infiltration was assessed by IHC and immunofluorescence (IF) staining of tissue sections. TMZ-induced pyroptosis was assessed by observation of morphological changes, WB and ELISA detection. Only GSDMD expression was upregulated in glioma compared with nontumor brain tissues both in the public datasets and in-house cohort. High GSDMD expression was significantly associated with WHO grade IV, IDH 1/2 wild-type and mesenchymal subtypes. Besides, high GSDMD expression was associated with shorter overall survival and could be used as an independent risk factor for poor outcomes in LGG and GBM. GO enrichment analysis and IHC validation revealed that GSDMD expression might participate in regulating macrophage infiltration and polarization. TMZ treatment induced the pyroptosis in GBM cells and GSDMD expression increased with after treating with TMZ in a time-dependent manner. Moreover, knocking down GSDMD obviously decreased IL-1β expression and reduced TMZ-induced pyroptosis in in vitro. GSDMD was a novel prognostic biomarker, as well as TMZ-treatment response marker in glioma.

## 1. Introduction

Over 65% of all malignant primary brain tumors are glioblastoma, formerly also referred to as glioblastoma multiforme (GBM) [1]. In general, the incidence rate of gliomas is about one sixth of all the brain tumors diagnosed annually, i.e., 17,000 new cases of GBM are diagnosed per year [2]. However, the median survival period is less than 2 years with malignant GBM progression, even with standard treatment (surgical resection, adjuvant radiotherapy, and chemotherapy) [3]. Due to their intratumoral heterogeneity and various mutual signatures (e.g., IDH mutation/1p19q co-deletion status, MGMT promoter methylation status, TERT promoter mutations) the chances for establishing a universal standard treatment of GBM are limited [4]. The poor prognosis and the difficulty in treatment owe to the alteration and regulation of genes. Thereby, gene-targeted therapy was supposed to be a relatively effective therapeutic tactic for patients with GBM.

The gasdermin gene family was first found to participate in causing several alopecia-like skin mutations in mice [5]. The gasdermins (GSDMs) constitute a protein superfamily classified by the gasdermin domain and include six members (GSDMA), GSDMB, GSDMC, GSDMD, GSDME (also known as DNFA5) and DFNB59 (also known as PJVK) [6]. The GSDM family was the foremost substrate of inflammatory caspases and execution of pyroptosis, a newly discovered programmed cell death process that occurs during several stress conditions, including cancers [7,8]. Recent studies have shown that the GSDM family was involved in cell-growth regulation, inflammatory response and chemotherapy response in cancers [9,10,11]. GSDMB expression was elevated in HER2-postive breast cancer and high GSDMB expression associated with poor prognosis [12]. While GSMDC can be cleaved by caspase-8 and PD-L1-regulated expression of GSDMC, which subsequently induced pyroptosis and facilitated tumor necrosis. Currently, no study is performed to investigate the role of the GSDM family in glioma. The expression pattern and their potential genetic role in glioma remains to be illustrated.

In this study, we used public glioma datasets and an in-house cohort to explore the expression pattern and prognostic role of GSDMs in lower-grade glioma (LGG) and GBM. GSDMD was identified as a novel marker independently associated with prognosis of LGG and GBM patients. Further analysis also revealed GSDMD might participate in regulating the infiltration of immune cells, especially for macrophages. Moreover, the GSDMD-activated pyroptosis-related pathway in GBM, which might be crucial in mediating temozolomide (TMZ) response in GBM. For the first time, this study elaborated the prognostic role and TMZ-treatment response marker of GSDMD in glioma. 

## 2. Methods

### 2.1. Glioma Tissues

A paraffin-embedded glioma tissue microarray that contained 132 glioma samples and 10 nontumor brain tissues was used. Six additional frozen glioma tissues and four nontumor brain tissues (all collected from patients with traumatic brain injury) were used. All specimens were obtained from hospitalized patients during March 2017 and December 2020 in the department of neurosurgery at in this study. None of the patients received any chemo- or radiotherapy before surgery. All patients signed informed consents and this study was approved by the institutional ethics committee of the faculty of medicine at Wuhan University’s Renmin Hospital [approval number: 2012LKSZ (010) H].

### 2.2. GlioVis Analysis

Oncomine platform (www.oncomine.org, accessed on 19 March 2021) was used to explore the mRNA expression of GSDMs genes in glioma. A *p* value less than 0.05 was regarded as statistical significance. The genomic alterations of GSDMs genes in TCGA-GBM and TCGA-LGG were identified by the cBioPortal platform (http://www.cbioportal.org/, accessed on 19 March 2021).GlioVis website (http://gliovis.bioinfo.cnio.es/, accessed on 19 March 2021) is an important platform for data visualization and analysis to explore brain tumors. Except for normalized gene expression data, there is also information on glioma molecular pathology and GBM subtypes. A total of six datasets were utilized in our research: TCGA-LGG, TCGA-GBM, TCGA-GBMLGG, CGGA, Gravendeel and Rembrandt. The datasets were gained from the GlioVis platform. The GSMD family was analyzed by Search Tool for the Retrieval of Interacting Genes (STRING) (http://string-db.org, accessed on 4 April 2021). The functional interactions of the GSDM family described in STRING were then combined with the functional annotation groups described in Metascape (http://metascape.org/, accessed on 4 April 2021).

### 2.3. Immunohistochemical (IHC) Staining and Evaluation 

IHC staining for all proteins was performed on FFPE human glioma samples. In short, slides were prepared by conventional dewaxing hydration procedures. Then, the slides were immersed immediately in the antigen repair solution at 95 °C for 10 min, and left to cool naturally. Triton-PBS (100×) was used for cells permeation and 1% BSA was then used for blocking. Slides were incubated with primary antibodies (GSDMD, No. 20770-1-AP,1:100, Proteintech Wuhan, Hubei, China; GSDME, No. 13075-1-AP, 1:200, Proteintech) and then recognized by HRP Goat anti-Rabbit IgG secondary antibodies (No. 15015, 1:500, Proteintech). The nuclei were stained with 3,3-diaminobenzobutyl (DAB) and then kept away from light for 10 min at room temperature. Finally slides covered with neutral balsam and scanned with an Olympus BX40 microscope (Tokyo, Japan).

The intensity and the percentage of immune-reactive cells were both evaluated. Staining intensity was scored as follows: no staining (0); faint yellow staining(1); intermediate positive (2); and brown staining (3). Staining of GSDMD and GSDME were scored by the percentage of positive cells (0, <10%; 1, 10–25%; 2, 26–50%; 3, 51–75%; 4, >75%). The final immunoreactive score (FIS) was calculated as: staining intensity × percentage of positive cells. We defined FIS (0–4) as low expression and FIS (6–12) as high expression. Two individuals were separately responsible for the assessment of the results of IHC staining.

### 2.4. Immunofluorescence Staining

Glioma tissue sections were dewaxed and dehydrated with gradient alcohol. Then sections were placed into EDTA antigen repair buffer (PH 8.0) for antigen repair in a microwave oven. The microwave oven was at medium power for 8 min and then low power for 7 min. Slides were naturally cooled for 5 mins and then placed into PBS (PH 7.4), and shaken and washed on a decolorizing shaker for 5 min three times. Autofluorescence quencher was added to tissue sections for 5 min, and washed with running water for 10 min. Add 1%BSA and incubate for 30 min. The blocking solution was removed, and the slices were laid flat in a wet box and incubated at 4 °C overnight. The slides were then covered with secondary antibody corresponded to the first antibody, and incubated for 50 min at room temperature in the dark. DAPI dye solution was added dropwise, and incubated at room temperature in the dark for 10 min. Finally, antifluorescence quenching liquid was added for sealing. The slices were observed under fluorescence microscope and the images were collected. DAPI’s ultraviolet excitation wavelength is 330–380 nm, its emission wavelength is 420 nm, and it emits blue light. The excitation wavelength of FITC is 465–495 nm, and the emission wavelength is 515–555 nm, giving off green light. CY3 has an excitation wavelength of 510–560, an emission wavelength of 590 nm, and emits red light.

### 2.5. Cells and Cell Culture

Glioma cell lines (U87 and U251) were purchased from the Cell Bank Type Culture Collection of the Chinese Academy of Sciences (Shanghai, China). Cell lines were all cultured with Dulbecco’s modified Eagle’s medium (DMEM) supplemented with 10% fetal bovine serum (Gibco, Invitrogen, Carlsbad, CA, USA) under a humidified atmosphere of 5% CO_2_ at 37 °C. Temozolomide was purchased from Selleck (https://www.selleck.cn/, Lot: CCRG 81045, accessed on 16 September 2020). U87 and U251 cells were treated with temozolomide at various concentrations (200, 400, 800 μM) for 24 h.

### 2.6. Western Blot

Cells were lysed in modified RIPA buffer, supplemented with protease inhibitors, then centrifuged at 12,000 rpm for 15 min. Protein concentrations were detected using a BCA protein quantitative kit. Cell lysates were mixed with loading buffer and heated for 5 min at 100 °C. Proteins were separated by SDS-PAGE. Next, wet electroblotting (BioRad, Hercules, CA, USA) was used to transfer the protein onto a poly-vinylidene fluoride (PVDF) membrane. PVDF membrane was blocked in 5% nonfat milk. for 1 hour. The incubation with primary antibody (overnight at 4 °C) and secondary antibody were implemented one after another. Western blot analyses were repeated three times.

### 2.7. Statistical Analysis

Data were expressed as means ± standard deviations (SD) or ± standard error of the mean. Significant differences between the means ± the standard deviation of two different groups were examined using a Student *t*-test; for more than two groups, one-way ANOVA was used. Spearman correlation analysis was used to examine the correlation analysis. The high- and low-expression groups were categorized by the gene expression level of the optimal cutoff value. The optimal cutoff was determined using the GlioVis platform. Differences in survival between groups were analyzed using Kaplan–Meier survival analysis with a log-rank significance test. Univariate and multivariate Cox regression models were performed to detect the prognostic elements. GraphPad Prism 8.0 software (GraphPad Inc., San Diego, CA, USA) was used to produce graphs.

## 3. Results

### 3.1. Transcriptional Level of GSDMs in Glioma

**Figure 1:**
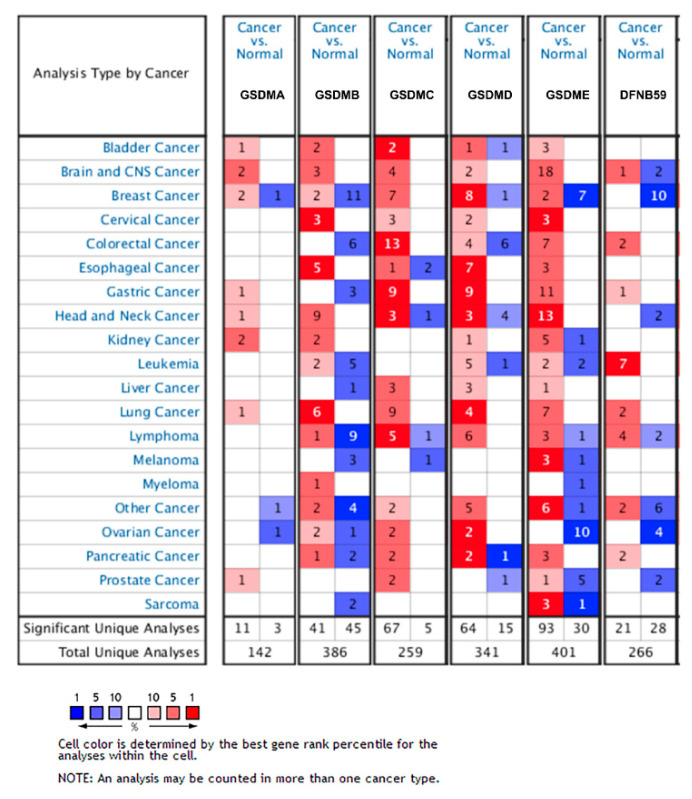
Transcriptional level of GSDMs in glioma. The mRNA and protein alteration of GSDMs in different types of cancers in Oncomine.

To obtain comprehensive information of GSDMs expression in glioma, we analyzed genetic alteration by using Oncomine and cBioportal. The results showed that GSDMs were all obviously elevated in brain and CNS cancer compared with normal tissues, except for DFNB59 (Figure 1). Using cBioPortal, we analyzed genetic alterations of the GSDMs and found relatively lower alteration frequency of GSDMs in glioblastoma. While the genetic alterations of GSDMD and GSDME in LGG reached a high frequency of 32% and 22%, respectively (Figure S1). 

### 3.2. GSMDs mRNA Expression Associated with Glioma WHO Grade

**Figure 2:**
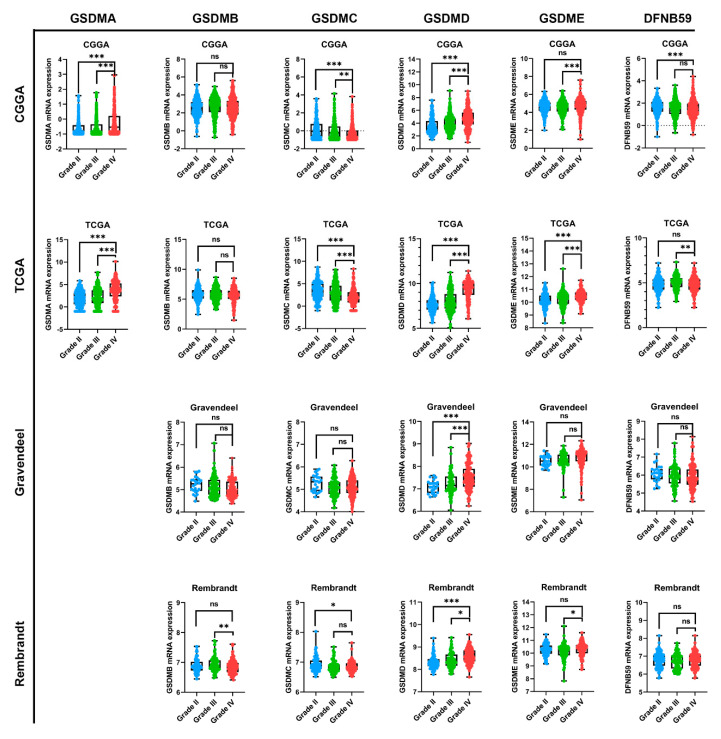
GSMDs mRNA expression associated with glioma WHO grade. Four public datasets, namely TCGA, CGGA, Gravendeel and Rembrandt were used to investigate GSDMs expression in glioma. All data were downloaded from the GlioVis platform. GSDMA expression was missing in Gravendeel and Rembrandt. The number of Grade I patients was too small and did not affect the final results. * *p* < 0.05, ** *p* < 0.01. *** *p* < 0.001. ns, no significance.

In order to further analyze GSDMs expression in glioma, we employed four public datasets, namely TCGA, CGGA, Rembrandt and Gravendeel. GSMDA gene was missing in Gravendeel and Rembrandt datasets. As results, we found that GSMDA expression was higher in glioblastoma compared with lower grade glioma (WHO I-III) in CGGA and TCGA (Figure 2). While the trends of GSDMB, GSMDC and DFNB59 expression in glioma were not consistent across four datasets. GBM expressed significantly higher levels of GSDMD mRNA than LGG, suggesting that GSDMD expression increased progressively with higher glioma grade in CGGA, TCGA, Gravendeel and Rembrandt (Figure 2). GSDME expression was obviously increased in GBM in CGGA, TCGA and Rembrandt, while there was no difference between LGG and GBM of GSDME expression in the Gravendeel dataset (Figure 2). 

### 3.3. Gene Ontology Enrichment Analysis

**Figure 3:**
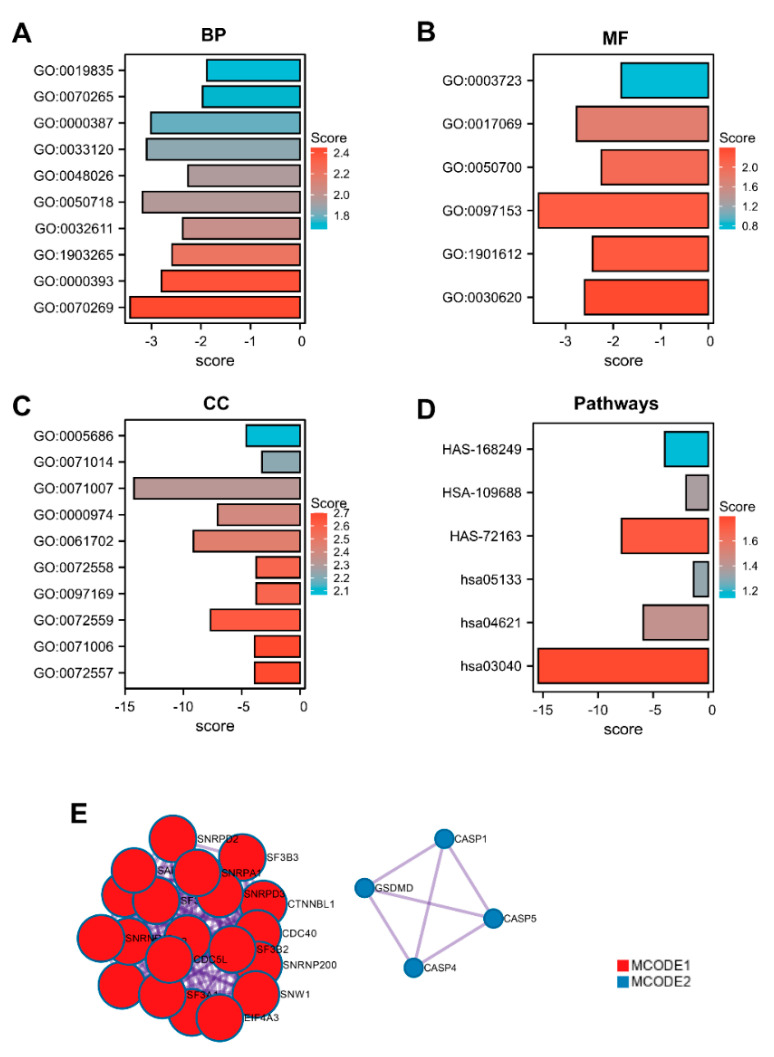
Gene ontology enrichment analysis. Proteins interacting with the GSDM family were screened using STRING (www.string-db.org, accessed on 4 April 2021). GO enrichment analysis significantly distributed GSDM−related genes into (**A**) biological processes; (**B**) molecular functions; (**C**) cellular component categories and (**D**) KEGG pathways prediction. (**E**). Analysis of protein−protein interaction networks was performed in Metascape (http://metascape.org/, accessed on 4 April 2021).

The functions of the GSDMs gene were projected by analyzing GO and KEGG enrichment in the STRING database. Our results revealed that the top five enriched GO-biological process terms of GSDMs were: GO:0070269 (pyroptosis), GO:0000393 (spliceosomal conformational changes to generate catalytic conformation), GO:1903265 (positive regulation of tumor necrosis factor mediated signaling pathway), GO:0032611 (interleukin-1 beta production) and GO:0050718 (positive regulation of interleukin-1 beta secretion) (Figure 3A). The enriched molecular function gene sets of GSDMs mainly involved in GO:0030620 (U2 snRNA binding), GO:1901612 (cardiolipin binding) and GO:0097153 (cysteine-type endopeptidase activity involved in apoptotic process) (Figure 3B). Significantly enriched GO terms related to cellular component were as follows: GO:0072557 (IPAF inflammasome complex), GO:0071006 (U2-type catalytic step 1 spliceosome) and GO:0072559 (NLRP3 inflammasome complex) (Figure 3C). In KEGG pathway analysis, hsa03040 (Spliceosome), hsa04621(NOD-like receptor signaling pathway) and hsa05133(Pertussis) were showed to be enriched significantly (Figure 3D). PPI analysis of GSDM-related genes described previously was performed in STRING, and two significant gene modules were selected using the MCODE application (Figure 3E). 

### 3.4. Prognostic Role of GSMDs in Glioma 

Initially, Kaplan–Meier curves were used to plot overall survival against optimal cutoff. We found that glioma patients with high GSMDA, GSDMD and GSDME expression were all associated with shorter overall survival time. While there was no significant difference for high and low expression of GSDMB, GSMDC and DFNB59 and overall survival in public datasets (Figure S2).

**Figure 4:**
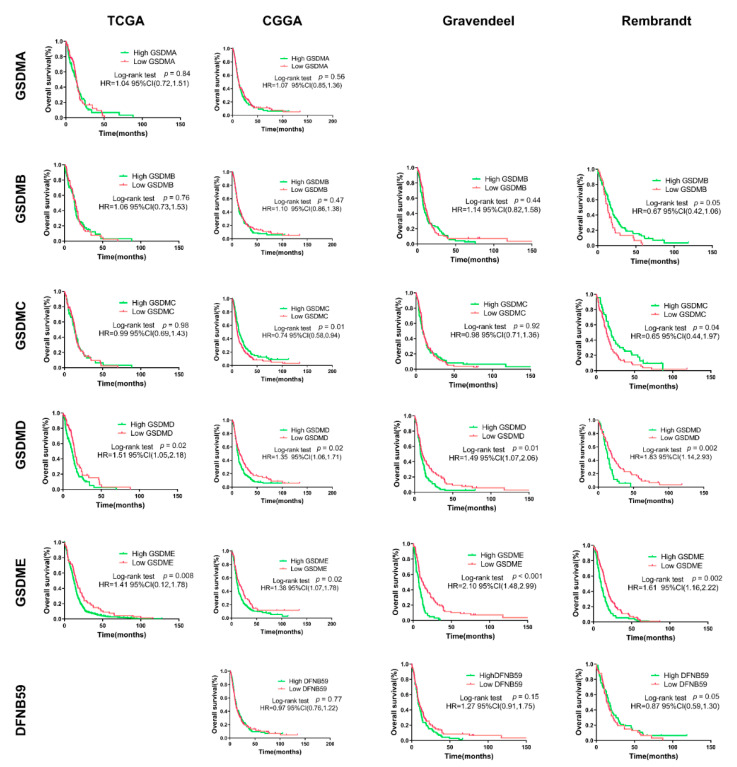
Prognostic role of GSMDs in glioma. Differences in survival between high and low expression in GBM patients were analyzed using Kaplan–Meier analysis with a log-rank significance test. All data were downloaded from GlioVis and optimal cutoff determined using GlioVis. GSDMA expression data was missing in Gravendeel and Rembrandt, while the TCGA-GBM dataset lacked DFNB59 expression. HR, hazard ratio.

Kaplan–Meier survival analyses were performed separately on LGG and GBM. As presented in Figure 4, GSDMA, GSDMB and DFNB59 expression could not obviously divide GBM patients into survival groups in the four public datasets. High GSDMC expression in GBM patients predicted favor prognosis in Rembrandt dataset, while no significant difference in survival time was observed between low and high GSDMC expression groups in TCGA, CGGA and Gravendeel. Besides, survival curve analysis revealed that GBM patients with high expression of GSDMD presented a higher percentage of overall survival than patients with low expression of GSDMD in TCGA, CGGA, Gravendeel and Rembrandt datasets. Importantly, GBM patients with high GSDME expression had shorter median survival than patients with low GSDME expression in the TCGA, CGGA, Gravendeel and Rembrandt datasets. 

For LGG, we found that higher GSDMC expression predicted longer survival time in TCGA and Gravendeel, while the opposite trend was observed in CGGA (Figure S2). There were no consistent trends in the Kaplan–Meier analysis of GSDMD expression in predicting prognosis of LGG patients. A significant difference between high and low GSDMD groups was only observed in the TCGA and Rembrandt datasets. While survival time of high GSDME expression was lower than that of low GSDME expression in LGG patients in three of the four public datasets (Figure S2).

To further reveal independent risk factors of poor prognosis in glioma patients, we performed multivariable Cox proportional hazard regression analysis. The results showed that WHO grade IV, IDH 1/2 wild-type, and high GSDME expression was independently associated with poor prognosis of glioma patients in TCGA (Table 1). While in the CGGA dataset, we found that age (>60 years), WHO grade IV, IDH 1/2 wild-type, high GSDMD and GSDME expression could be used independently to predict the prognosis of gliomas patients (Table 2).

### 3.5. In-House Cohort Validation

**Figure 5:**
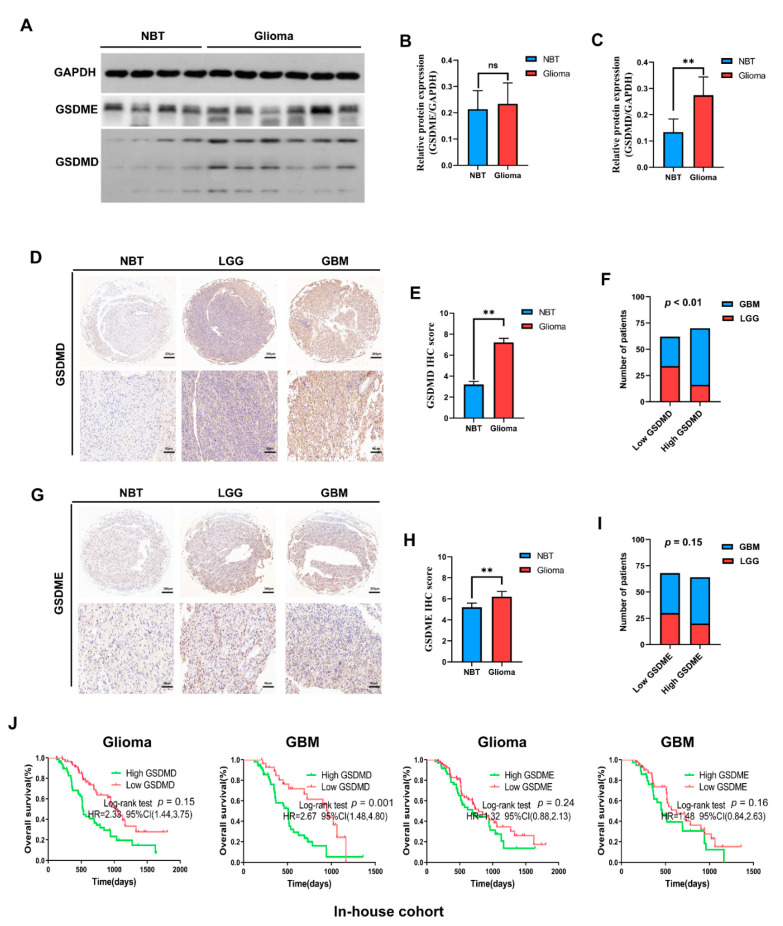
In-house cohort validation (**A**–**C**) Western blot detection of GSDMD and GSDME expression in glioma tissues and nontumor brain tissues (NBT). GAPDH was used as internal reference. (**D**–**F**) IHC staining of GSDMD in NBT, lower-grade glioma (LGG) and glioblastoma (GBM). (**G**–**I**) IHC staining of GSDMD in NBT, LGG and GBM. (**J**) Kaplan–Meier analysis was employed to assess the association between GSDMD/GSDME expression and the prognosis of glioma patients. ** *p* < 0.01. ns, no significance. HR, hazard ratio.

Results presented above indicated GSDMD and GSMDE might play crucial roles in the malignancy process of glioma, and be potential biomarkers in predicting prognosis of GBM. In order to illustrate expression pattern and prognostic role of GSDMD and GSDME in glioma, we conducted further experimental validations. Results of Western blot analysis revealed that GSDMD, not GSDME was elevated in glioma tissues when compared with non-brain tumor tissues (Figure 5A–C). Simultaneously, IHC analysis showed that GSDMD expression was significantly higher in glioma tissues and correlated with higher glioma WHO grade (Figure 5D–F). Interestingly, measurement of GSDME expression using IHC showed that GSDME expression was significantly higher in glioma tissues, but showed no correlation with higher glioma grade (Figure 5G–I). 

Mean and median follow-up times for the in-house cohort were 621.80 and 516 days, respectively. We found that higher GSDMD expression predicted shorter survival time of glioma and GBM patients, while no significant difference in survival time was observed between low and high GSDME expression groups in our cohort (Figure 5J). 

### 3.6. High GSDMD Expression Associated with IDH Wildtype, 1p19q Codeletion and Subtypes

**Figure 6:**
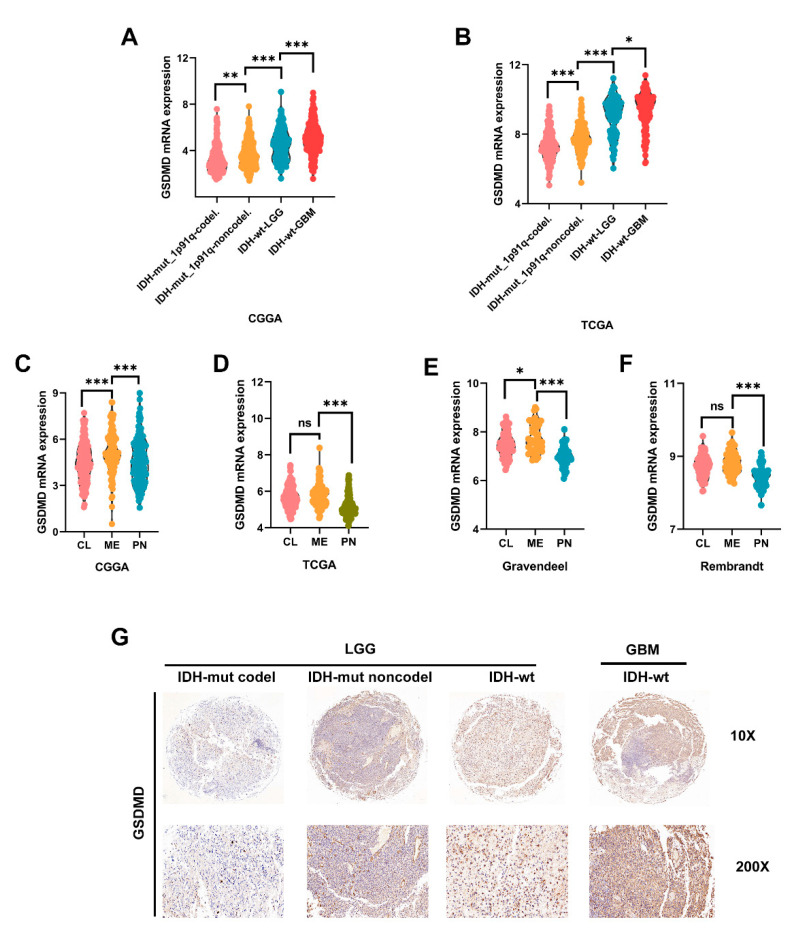
High GSDMD expression associated with IDH wildtype, 1p19q co-deletion and subtypes (**A**,**B**) GSDMD expression in glioma tissues with different molecular characteristics. IDH and 1p19q status of glioma in CGGA and TCGA datasets were all downloaded from GlioVis. IDH, isocitrate dehydrogenase. WT, wildtype. Mut, mutant. (**C**–**F**) GSDMD expression in different subtypes of GBM was analyzed in CGGA, TCGA, Gravendeel and Rembrandt datasets. CL, Classical; ME, mesenchymal; PN, proneural. (**G**) IHC staining was used to detect GSDMD expression in glioma tissues (in-house cohort) with different IDH and 1p19q status. * *p* < 0.05. ** *p* < 0.01. *** *p* < 0.001. ns, no significance.

Two mutation (IDH1/2 mutations and 1p19q co-deletion) tests have become a part of the routine diagnosis and classification of gliomas [13]. In CGGA and TCGA, we found that GSDMD expression was mostly enriched in GBM patients with IDH-wt. LGG patients with IDH-wt also presented higher GSDMD expression than those with IDH-mut, with or without 1p19q co-deletion (Figure 6A,B). The in-house cohort’s glioma tissues were partially gene sequenced. The association between GSDMD and clinical pathological parameters of was presented in Table 3. To confirm our findings, we analyzed IHC staining and found that LGG with IDH-mut with or without 1p19q co-deletion had lower GSDMD expression than patients with IDH-wt. GBM patients with IDH-wt had highest GSDMD expression among four groups (Figure 6G).

Molecular subtypes (classical, mesenchymal and proneural) were key features for classification and prognosis of glioma [14,15]. Glioma cells with a mesenchymal (ME) phenotype, in contrast to other subtype cells, showed higher expression of invasion-related proteins to improve migration and invasion [16,17]. In this study, GSDMD was significantly higher in ME subtype GBM than in other subtypes in CGGA and Gravendeel (Figure 6C–E). While in TCGA and Rembrandt, GSDMD level was slightly elevated in ME GBM compared with the classical subtype. However, the difference was not significant (Figure 6D–F).

### 3.7. GSDMD Associated with Immune Cell Infiltration in Glioma

**Figure 7:**
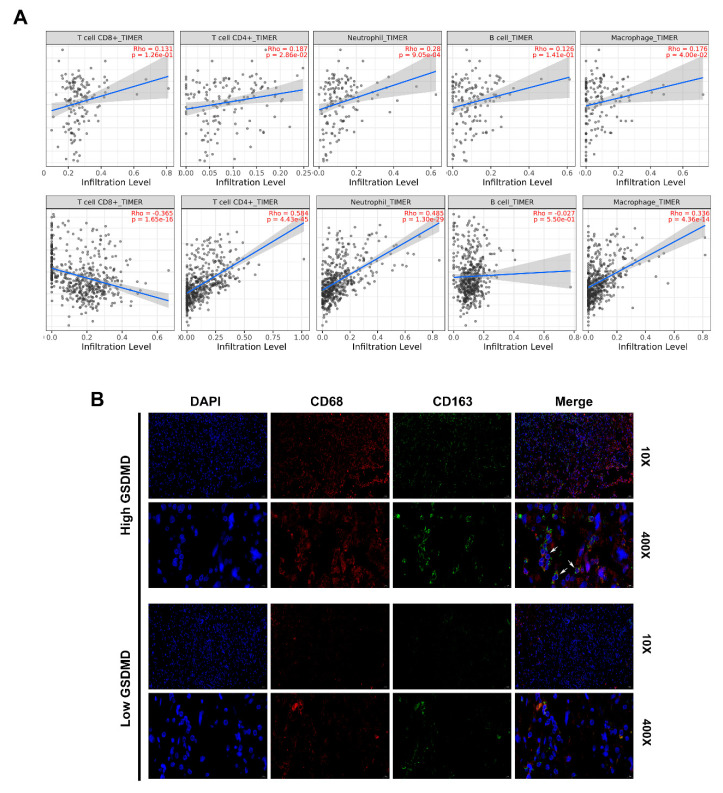
GSDMD associated with immune cell infiltration in glioma (**A**). TIMER was used to assess the association between GSDMD expression and immune cell infiltration in LGG and GBM. (**B**). Immunofluorescence double-staining for CD68 and CD163 in glioma tissues with high or low GSDMD expression. White arrows indicate CD68+/CD163+ cells. Nuclei were detected by DAPI staining.

Given the important role of the immune cells infiltration and functions in promoting glioma malignancy, it is the key to explore novel targets in cancer immunotherapy. We evaluated the correlation between GSDMD expression and the immune infiltration levels from TIMER. The level of GSDMD expression correlated with high levels of immune infiltration of CD4+ T cells, macrophage and neutrophil both in LGG and GBM (Figure 7A). Furthermore, we found that GSDMD expression positively correlated with CD8A/CD8B/CD3D/CD3E (T cell markers), CD86/CD79A/CSF1R (B cell), CCL2/CD68 (monocyte), CD163/IRF5 (macrophage) and ITGAM/CCR7 (neutrophil) both in TCGA and CGGA. Besides, T cell exhaustion marker genes (HAVCR2, CTLA4, LAG3, PDCD1 and BTLA) showed strong correlations with GSDMD expression (Table 4). 

Then we used we single-cell sequencing datasets from the TISCH database and found that GSDMD was closely associated with infiltration of monocyte/macrophage in gliomas (Figure S3A,B). Further analysis revealed that glioma tissues with high GSDMD expression had abundant macrophage cell (CD68+/CD163+ double positive) infiltration by using IF staining (Figure 7B). Then positive correlations were shown between GSDMD and CD163 expression in glioma tissues by IHC (Figure 3C,D). Our results gave a hint that GSDMD might be novel maker associated with macrophage infiltration in glioma. 

### 3.8. GSDMD Mediated TMZ-Induced Pyroptosis in Glioma

**Figure 8:**
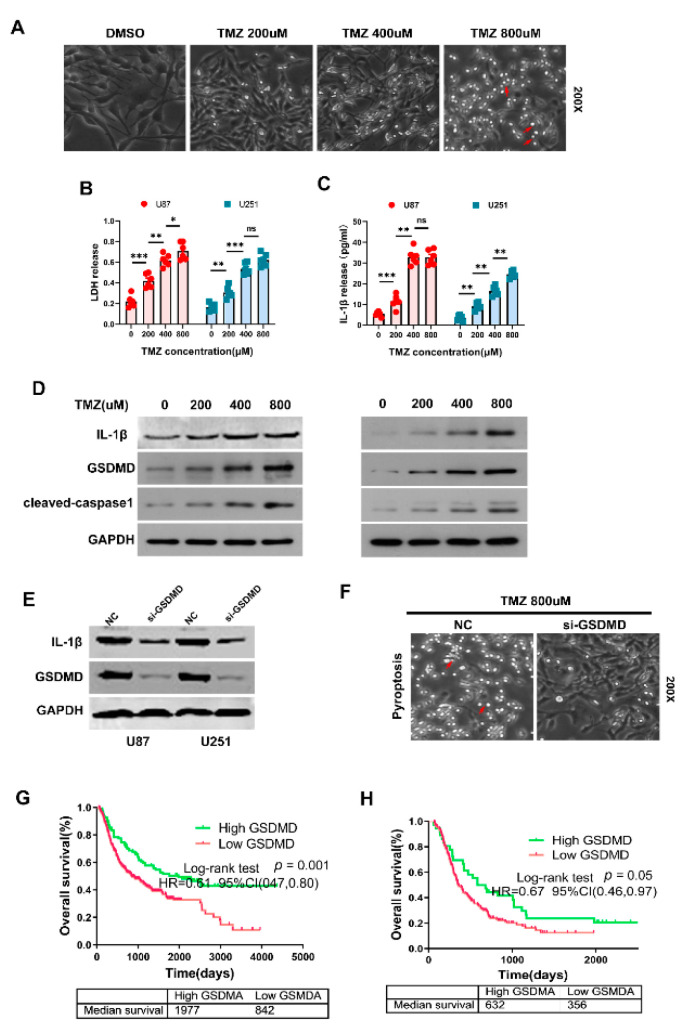
GSDMD mediated TMZ-induced pyroptosis in glioma. (**A**) Morphological changes in U251 cells treated with different concentrations of TMZ. Cells presented with large bubbles emerging from the plasma membrane (red arrows). (**B**,**C**) ELISA kits were used to measure the level of LDH and IL-1β after treatment with TMZ. * *p* < 0.05. ** *p* < 0.01. *** *p* < 0.001. ns, no significance. (**D**) Expression of GSDMD, cleaved caspase 1 and IL-1β were detected using WB in U251 and U87 cells treated with TMZ. GAPDH was used as internal reference. (**E**,**F**) After transfection of siRNA against GSDMD, the expression of GSDMD and IL-1β were examined by WB. Morphological changes were observed (red arrows). (**G**,**H**) Prognostic role of GSDMD in predicting TMZ response in glioma patients in CGGA. HR, hazard ratio.

Temozolomide (TMZ) is the first-line therapy for glioma. We tried to found association between GSDMD expression and TMZ response in glioma. We treated glioma cells U87 and U251 with different concentrations of TMZ and the results showed that glioma cells treated with TMZ presented with large bubbles emerging from the plasma membrane, which became more noticeable as the drug concentration increased (Figure 8A). Significant increase in the LDH and IL-1β level in the supernatants were detected when glioma cells treated with TMZ using ELISA kits (Figure 8B,C). Moreover, we found that GSDMD, IL-1β and cleaved-caspase-1 expression were all significantly elevated after treating cells with different concentrations of TMZ (Figure 8D). GSDMD expression increased after treating with TMZ in a time-dependent manner (Figure 8D and Figure S4). Knocking down GSDMD obviously decreased IL-1β expression and reduced TMZ-induced pyroptosis in vitro (Figure 8E,F and Figure S4). These results strongly indicated that GSDMD might participate in mediating TMZ induced pyroptosis in glioma. Then we investigated the prognostic role of GSDMD in predicting TMZ response in glioma patients. Our results showed that high GSDMD expression in glioma patients who received chemotherapy predicted favor prognosis in CGGA dataset (Figure 8G,H). The median survival time of GBM patients with high GSDMD expression showed a trend to those with low GSDMD expression (632 days vs. 356 days), but was not statistically significant (*p* = 0.05) (Figure 8G,H).

## 4. Discussion

Pyroptosis is an inflammation-dependent type of programmed cell death, which is mediated by inflammasomes [18]. Distinct from apoptosis, pyroptosis needs the participation of the gasdermin family as executioners to induce cell swelling with large bubbles blowing from the plasma membrane. Activated GSDMD/GSDME protein leads to the formation of membrane pores and mediates the maturation and release of IL-1β and IL-18, which subsequently induces strong inflammatory response in tumor microenvironment [19]. Glioma is a highly proinflammatory tumor. Human GBM cell lines and cultured primary glioma cells can secrete a large amount of IL-1β, IL-6 and IL-8, and promote tumor growth in an autocrine or paracrine way [20]. Increasing numbers of studies show that inflammation-related genes’ regulatory network exerts essential functions in modulating the development of glioma [21,22]. This study, for the first time, found that GSDMD was overexpressed in glioma and increased significantly as glioma grade developed. Besides, high GSDMD expression predicted unfavorable prognosis of LGG and GBM patients and was an independent risk factor associated with poor outcome. GSDMD expression in glioma significantly correlated with clinical parameters, such as IDH1/2 wildtype, 1p19q non-co-deletion and mesenchymal subtype. These results strongly indicated that GSDMD, a member of the gasdermin family, was a novel oncogene and could be used as a prognostic biomarker both in LGG and GBM. 

Moreover, GSDMD expression was obviously elevated in TMZ-treated glioma cells and became more noticeable as the drug concentration increased. Knocking down GSDMD expression dramatically reduced TMZ induced pyroptosis and decreased IL-1β and LDH expression. Taken together, these findings gave us a hint that glioma cells with high GSDMD expression might present with increasing sensitivity to chemotherapeutic drugs and decreasing drug resistance. 

Indeed, expression of gasdermins had been investigated in several solid tumors. GSDMB expression and not the expression of other GSDMs, was significantly elevated in HER-2 positive breast cancer and associated with poor survival. Besides, nearly 65% of investigated samples presented with GSDMB amplification and overexpression [23]. GSDMC expression was found to be obviously upregulated in lung adenocarcinoma (LUAD) tissues by using several bioinformatics platforms and PCR detection. LUAD patients with high GSDMC expression had worse first progression and shorter overall survival time than those with low GSDMC expression [24]. Our study shared a similar methodology with a previous study [24] that evaluated the GSDM family’s effect on expression and prognosis of cancer using integrative bioinformatics analyses and in-house validation. In this study, we found for the first time that GSDMD expression was significantly elevated in glioma tissues and elevated as glioma grade increased both in four public datasets and in-house validation using WB and IHC staining. Patients with high GSDMD expression had poor prognosis of LGG and GBM, which indicated that GSDMD could be used as prognostic biomarker both in LGG and GBM. Notably, GSDMD, an important mediator of pyroptosis, was seldom investigated in cancer. One study revealed that high GSDMD expression was positively correlated with larger tumor size, advanced TNM stages and also poor prognosis in LUAD [25]. Consistent with the role of GSDMD in LUAD and osteosarcoma [26], our evidence shows that GSDMD was a novel oncogene and prognostic biomarker for LGG patients and GBM patients. 

The 2016 WHO classification generated a combination of the histological and molecular characteristics of glioma, including IDH-1 mutation and 1p/19q co-deletion (1p19q co-del) [27]. IDH wild-type glioma has a worse prognosis than their mutant counterparts and shows more malignant biological behaviors, such as invasion, metastatic characteristics and chemoresistance [28,29]. IDH-mut with or without 1p19q co-deletion glioma patients had lower GSDMD expression that those with IDH1-wt, which indicated strong association between GSDMD and molecular features and therefore with its malignancy. IDH has an important role in cell metabolism, which is a hallmark of epithelial to mesenchymal transition (EMT) in GBM [30,31]. In our study, we found GSDMD expression was obviously elevated in mesenchymal (ME) subtypes and its expression significantly correlated with ME-related genes expression. However, whether GSDMD has important functions in mediating the IDH-induced EMT process in glioma is not well established. Further validation of molecular biology experiments is still needed. Actually, IDH1/2 status and the ME transition of glioma cell inevitably affect the components of tumor immune microenvironment, especially the components of lymphocytes [32,33]. GBM patients with ME features had higher reaction of M2 macrophage gene signature than M1 macrophage or neutrophil gene signatures. Deactivated NF1 gene (somatic mutations or genomic copy loss) is a ubiquitous characteristic in ME GBM. NF1 deficiency in GBM significantly reduced the infiltration of tumor- associated macrophages [34]. High GSDMD expression correlated with greater infiltration of neutrophil/macrophage in LGG and GBM, which revealed the important role of GSDMD in remodeling glioma microenvironment and promoting tumor progression. Although the results of IHC and IF validation could not provide sufficient evidence to support the direct relationship between GSDMD and immune infiltration, they provided reasonable assumptions for subsequent experiments.

GSDMD, a key pore-forming gasdermin protein, was found to release the activated cytokines and to induce pyroptotic cell death [35,36]. Recent studies had revealed that GSDMD was necessary for the maturation and release of IL-1β, which might be regulated by inflammasome activation, such as NLRP3, NLRC4 or NLRP1 inflammasomes [37,38]. Inflammasome/GSDMD/IL-1β axis has not been investigated in glioma. NLRP3/GSDMD/IL-1β may act as an important regulator of inflammatory responses, which not only modulates gliomagenesis but also GBM therapy response. TMZ has been used as a first-line agent for the treatment of glioma, but up to 50% patients were reported to have resistance to TMZ [39,40]. Glioma cells may become resistant to TMZ by evading apoptosis. Inducing the transformation from apoptosis to pyroptosis may be the key to overcome chemotherapy resistance. Lu Wang et al. found that metformin obviously induced GSDMD-mediated pyroptosis, which could be abolished by PELP1 overexpression [41]. A previous study reached a conclusion that pyroptosis-induced drugs could reverse or partially eliminate chemoresistance and might serve as an alternative treatments for malignant tumors [41]. For the first time, this study elaborated the fact of TMZ-induced pyroptosis and the crucial role of GSDMD in mediating TMZ-induced pyroptosis. During the treatment of glioma, TMZ could be selected according to the expression levels of GSDMD, which was obviously upregulated in GBM, thereby increasing the sensitivity to chemotherapeutic drugs and reducing drug resistance. Treatment with TMZ can induce the upregulation of GSDMD expression in GBM, causing pyroptosis and making these cells more sensitive to chemotherapeutic drugs. This study sheds new light on chemoresistance of glioma treatments.

## 5. Conclusions

GSDMD is a novel oncogene in glioma and strongly associated with tumor malignancy. GSDMD could be used as an independent prognostic biomarker, as well as TMZ-treatment response marker in glioma.

## Figures and Tables

**Table 1:** Univariate analysis and multivariate COX analysis of clinical prognostic parameters of all glioma in the TCGA dataset.

Variables	Univariate Cox Regression		Multivariate Cox Regression	
	HR (95%CI)	*p* Value	HR (95%CI)	*p* Value
Age (>60 years vs. ≤60 years)	2.54 (1.77–3.66)	<0.001	2.31 (1.62–3.30)	<0.001
Gender (Female vs. male)	0.94 (0.69–1.30)	0.72	-	-
WHO Grade (IV vs. I-III)	0.41 (0.27–0.64)	<0.001	0.37 (0.25–0.56)	<0.001
IDH status (wild-type vs. mutant)	3.99 (2.29–6.95)	<0.001	0.43 (0.23–0.82)	0.01
MGMT promoter (methy vs. unmethy)	1.31 (0.92–1.88)	0.13	-	-
Subtypes (ME vs. others)	0.46 (0.23–0.92)	0.03	-	-
GSDMA expression	0.98 (0.92–1.06)	0.72	-	-
GSDMB expression	1.12 (0.96–1.31)	0.17	-	-
GSDMC expression	0.96 (0.88–1.05)	0.40	-	-
GSDMD expression	1.10 (0.92–1.30)	0.34	-	-
GSDME expression	1.45 (1.08–1.93)	0.01	1.32 (1.03–1.70)	0.03
DFNB59 expression	0.96 (0.88–1.05)	0.06	-	-

**Table 2:** Univariate analysis and multivariate COX analysis of clinical prognostic parameters of all glioma in the CGGA dataset.

Variables	Univariate Cox Regression		Multivariate Cox Regression	
	HR (95%CI)	*p* Value	HR (95%CI)	*p* Value
Age (>60 years vs. ≤60 years)	0.96 (0.69–1.33)	0.81	-	-
Gender (Female vs. male)	0.98 (0.78–1.23)	0.78	-	-
WHO Grade (IV vs. I–III)	3.20 (2.40–4.24)	<0.001	3.25 (2.37–4.10)	<0.001
IDH status (wildtype vs. mutant)	2.47 (1.85–3.30)	<0.001	2.32 (1.75–3.08)	<0.001
GSDMA expression	0.92 (0.74–1.15)	0.45	-	-
GSDMB expression	1.01 (0.99–1.03)	0.56	-	-
GSDMC expression	0.96 (0.99–1.14)	0.11	-	-
GSDMD expression	0.99 (0.99–1.00)	0.02	0.99 (0.99–1.00)	0.03
GSDME expression	1.01 (1.00–1.02)	0.003	1.01 (1.00–1.02)	0.002
DFNB59 expression	0.99 (0.92–1.06)	0.76	-	-

**Table 3:** Comparison of clinical characteristics in different GSDMD expression groups among glioma patients.

Factors	GSDMD Expression		
	Low	High	*p* Value
Age (mean ± SD)	55.82 ± 10.90	53.57 ± 13.16	
Gender			0.46
Female	27	35	
Male	35	35	
Karnofsky score			0.15
>80	43	40	
≤70	19	30	
Grade			0.01
I	3	1	
II	11	3	
III	20	6	
IV	28	52	
IDH1/2 status			<0.01
Mutant	22	6	
Wildtype	7	15	
Chemotherapy	35	46	0.28
Radiotherapy	42	51	0.52

The chi-square test was used.

**Table 4:** Correlation between GSDMD expression and markers of immune cells.

Immune Cells	TCGA				CGGA		
	Markers	Cor	95%CI	*p* Value	Cor	95%CI	*p* Value
CD8+ T cell	CD8A	0.42	0.35–0.48	<0.0001	0.25	0.17–0.32	<0.0001
	CD8B	0.41	0.35–0.48	<0.0001	0.47	0.41–0.53	<0.0001
T cell	CD3D	0.62	0.57–0.67	<0.0001	0.63	0.58–0.68	<0.0001
	CD3E	0.65	0.60–0.70	<0.0001	0.44	0.37–0.50	<0.0001
B cell	CD86	0.60	0.55–0.65	<0.0001	0.46	0.40–0.52	<0.0001
	CD79A	0.30	0.22–0.36	<0.0001	0.44	0.37–0.50	<0.0001
	CSF1R	0.38	0.31–0.45	<0.0001	0.26	0.19–0.33	<0.0001
Monocyte	CCL2	0.56	0.50–0.61	<0.0001	0.31	0.24–0.38	<0.0001
	CD68	0.64	0.59–0.68	<0.0001	0.45	0.39–0.51	<0.0001
	NOS2	0.15	0.07–0.23	<0.0001	0.05	0.00–0.13	0.1561
macrophage	CD163	0.52	0.46–0.58	<0.0001	0.42	0.35–0.48	<0.0001
	IRF5	0.63	0.58–0.67	<0.0001	0.44	0.37–0.50	<0.0001
	PTGS2	0.26	0.19–0.33	<0.0001	−0.07	(−0.15)–0.01	0.0685
	CEACAM8	0.08	0.00–0.15	0.0509	-	-	-
	MS4A4A	0.55	0.49–0.60	<0.0001	0.38	0.32–0.45	<0.0001
Neutrophil	ITGAM	0.53	0.47–0.58	<0.0001	0.43	0.36–0.49	<0.0001
	CCR7	0.44	0.38–0.50	<0.0001	0.21	0.13–0.28	<0.0001
	KIR2DL1	0.04	0.00–0.12	0.2825	-	-	-
	KIR2DL3	0.13	0.05–0.20	0.0006	-	-	-
	KIR2DL4	0.35	0.28–0.41	<0.0001	-	-	-
T cell exhaustion	PDCD1	0.57	0.50–0.62	<0.0001	0.46	0.40–0.52	<0.0001
	CTLA4	0.50	0.44–0.56	<0.0001	0.44	0.37–0.50	<0.0001
	LAG3	0.32	0.24–0.38	<0.0001	0.60	0.54–0.64	<0.0001
	HAVCR2	0.64	0.59–0.68	<0.0001	0.34	0.27–0.41	<0.0001
	BTLA	0.35	0.28–0.42	<0.0001	0.25	0.18–0.32	<0.0001

## Data Availability

The data that support the findings of this study are available from the corresponding author upon reasonable request.

## Supplementary Materials

The following are available online at https://www.mdpi.com/article/10.3390/cancers13225620/s1, Figure S1: Genetic alteration-Gasdermins-TCGA, Figure S2: Gasdermins-Glioma-LGG, Figure S3: Association between GSDMD and macrophage infiltration, Figure S4: Original Western Blot Images.

## Abbreviations

CL	classical
DMEM	Dulbecco’s modified Eagle’s medium
EMT	mesenchymal transition
ELISA	enzyme linked immunosorbent assay
GBM	glioblastoma multiforme
GSDMs	gasdermins
HER-2	human epidermal growth factor receptor 2
IHC	immunohistochemistry
IF	immunofluorescence
IL-1β	interleukin-1 beta
IDH	isocitrate dehydrogenase
LUAD	lung adenocarcinoma
LGG	lower grade glioma
ME	mesenchymal
PVDF	poly-vinylidene fluoride
PN	proneural
STRING	Search Tool for the Retrieval of Interacting Genes
TMZ	temozolomide
WB	Western blot
