# Supplementary material for: Gasdermin D Is a Novel Prognostic Biomarker and Relates to TMZ Response in Glioblastoma
Source: Cancers (Basel). 2021 Nov 10;13(22):5620. doi: 10.3390/cancers13225620 (PMC8616249; doi:10.3390/cancers13225620)
Supplement: Supplementary file 1 [file cancers-13-05620-s001.zip › cancers-1439927 supplementary.pdf]

Supplementary Files

Gasdermin D Is a Novel Prognostic Biomarker and Relates with TMZ Response in Glioblastoma

Junhui Liu, Lun Gao, Xiaonan Zhu, Rongxin Geng, Xiang Tao, Haitao Xu and Zhibiao Chen

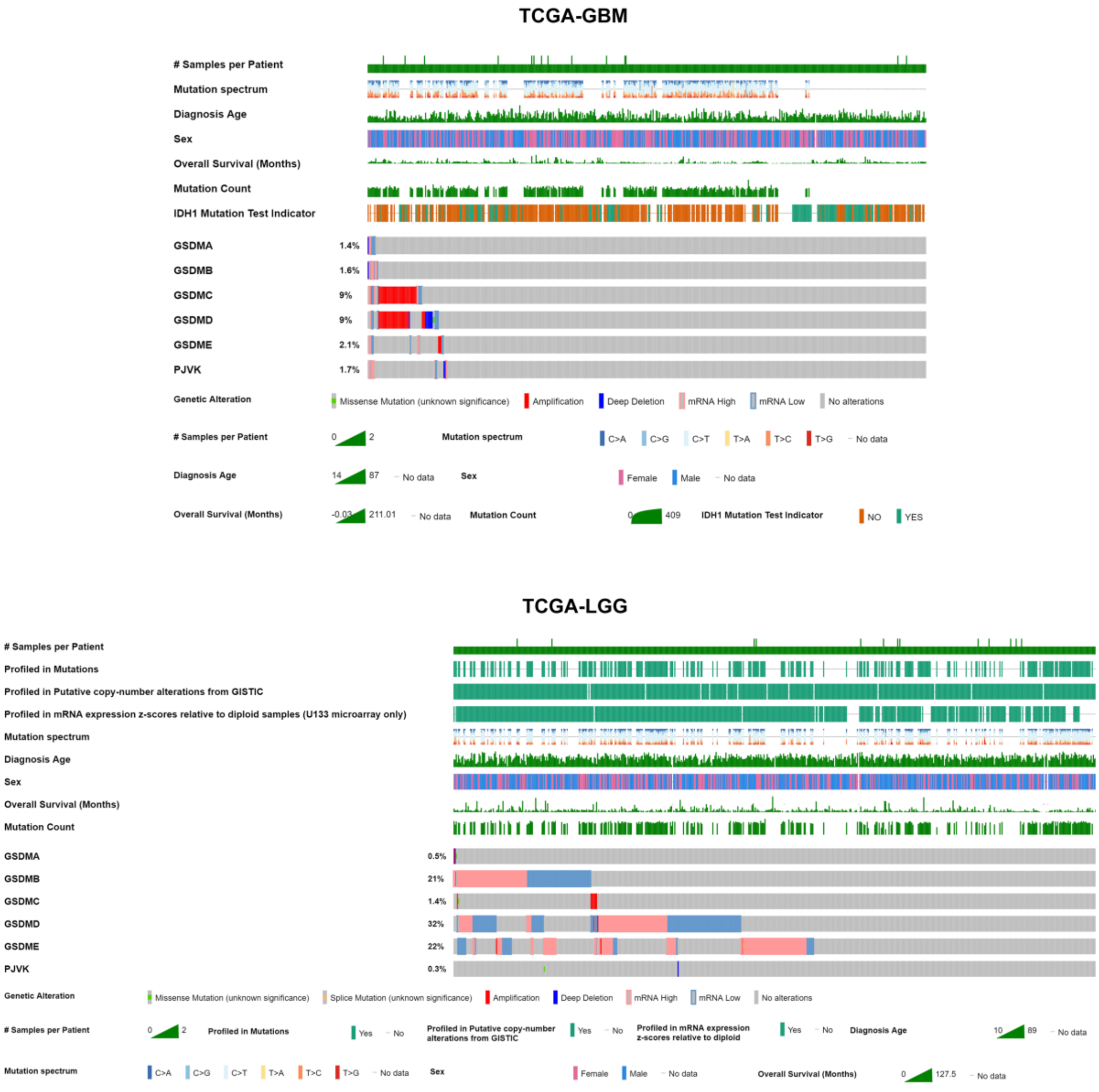

Figure S1. Genetic alteration-Gasdermins-TCGA.

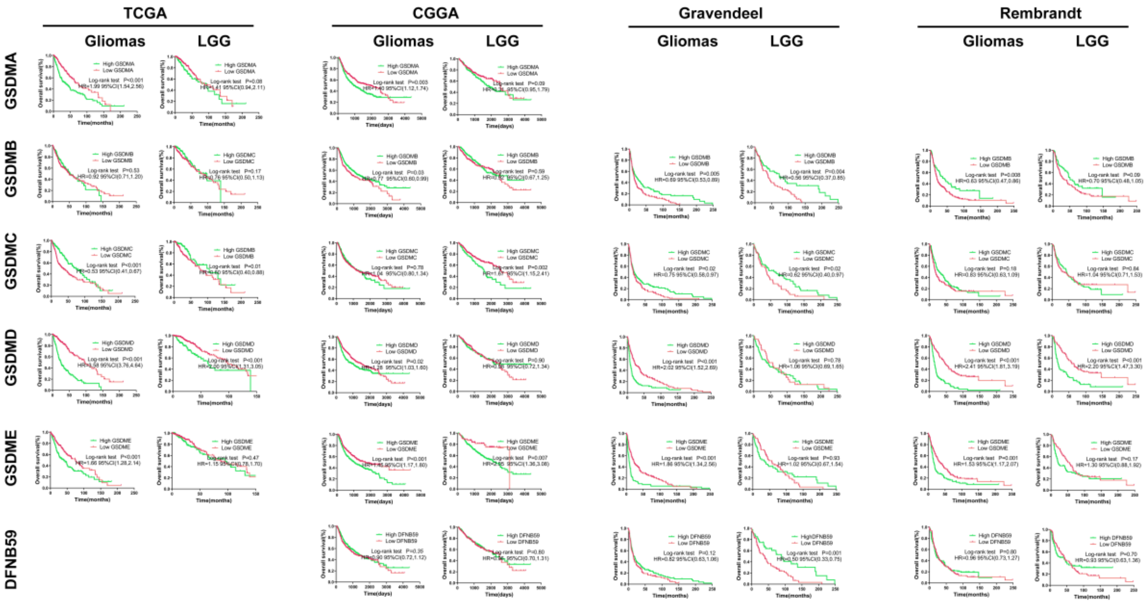

Figure S2. Gasdermins-Glioma-LGG.

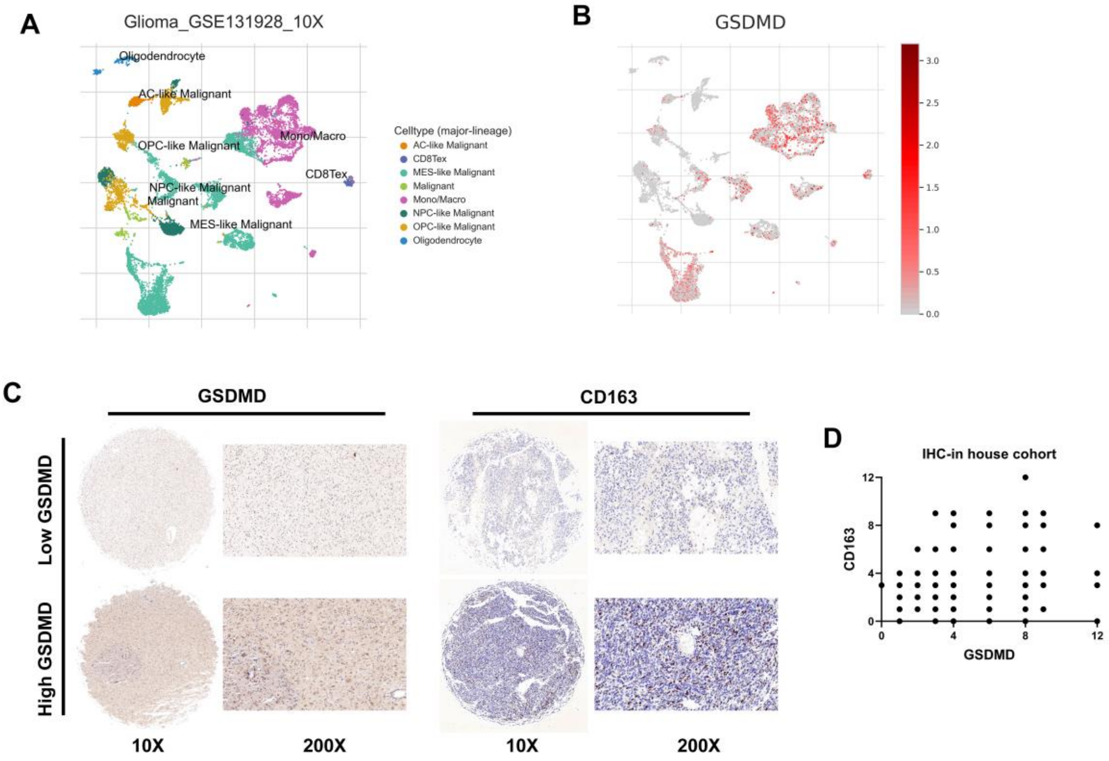

Figure S3. Association between GSDMD and macrophage infiltration. (**A,B**)**.** Association between GSDMD and immune cells infiltrition.Single-cell sequencing dataset (GSE131928_10X) from the TISCH database was used (**C,D**)**.** Correlation between GSDMD and M2 macrophage marker CD163 in human glioma tissues.

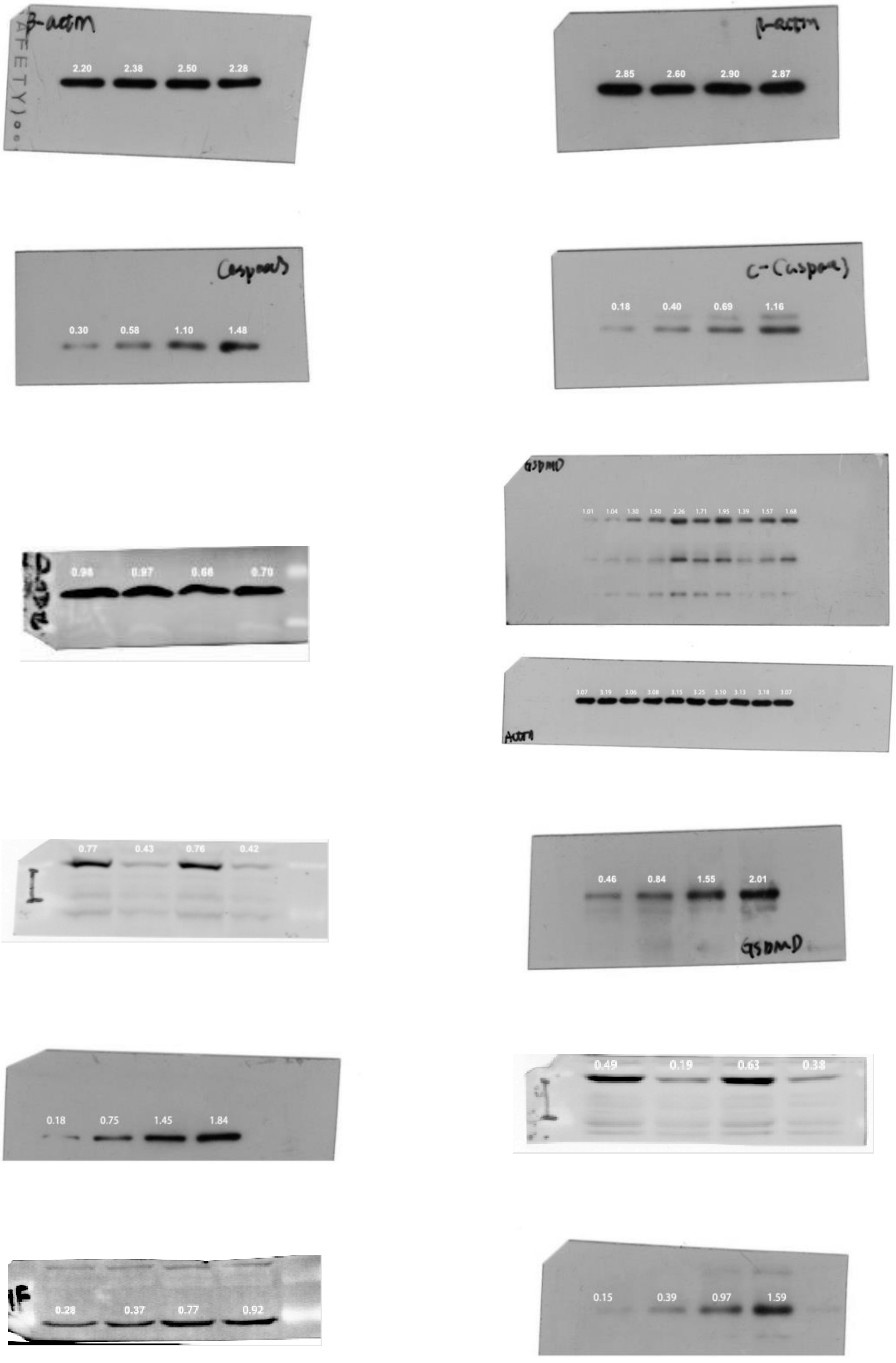

Figure S4. Original Western Blot Images.
